# Intron Retention in the Alternatively Spliced Region of RON Results from Weak 3’ Splice Site Recognition

**DOI:** 10.1371/journal.pone.0077208

**Published:** 2013-10-14

**Authors:** Lindsay D. Smith, Christian M. Lucas, Ian C. Eperon

**Affiliations:** Department of Biochemistry, University of Leicester, Leicester, United Kingdom; International Centre for Genetic Engineering and Biotechnology, Italy

## Abstract

The RON gene encodes a tyrosine kinase receptor for macrophage-stimulating protein. A constitutively active isoform that arises by skipping of exon 11 is expressed in carcinomas and contributes to an invasive phenotype. However, a high proportion of the mRNA expressed from the endogenous gene, or from transfected minigenes, appears to retain introns 10 and 11. It is not known whether this represents specific repression or the presence of weak splicing signals. We have used chimeric pre-mRNAs spliced *in vitro* to investigate the reason for intron retention. A systematic test showed that, surprisingly, the exon sequences known to modulate exon 11 skipping were not limiting, but the 3’ splice site regions adjacent to exons 11 and 12 were too weak to support splicing when inserted into a globin intron. UV-crosslinking experiments showed binding of hnRNP F/H just 5’ of these regions, but the hnRNP F/H target sequences did not mediate inhibition. Instead, the failure of splicing is linked to weak binding of U2AF65, and spliceosome assembly stalls prior to formation of any of the ATP-dependent complexes. We discuss mechanisms by which U2AF65 binding is facilitated *in vivo*.

## Introduction

RON is a universally expressed tyrosine kinase receptor for macrophage stimulating protein (MSP) [1]. MSP-dependent activation of RON induces downstream signalling pathways involved in wound healing, liver regeneration, bone resorption, embryogenesis and the immune response [2-7]. There are eight known variants of RON including Δ170, Δ165, Δ160, Δ155, Δ110, Δ55, Δ85 and Δ90, all of which arise through splicing alterations [8]. Some RON isoforms, such as RON Δ165, Δ160 and Δ155, are constitutively active. As with over-expression, constitutive activation confers an invasive and motile phenotype on epithelial cells and promotes metastasis (EMT) [9-13]. In particular, it results in the break-down of cell-cell contacts, promoting cell mobility and matrix invasion [14,15] and the progression of tumours [8,16]. Inappropriate RON signalling has been implicated in pancreatic [17], brain [16], colorectal [18], mammary [19], gastric [18], ovarian [20], hepatocellular [21], prostate [22], urinary [23] and renal [24] carcinomas. 

**Figure 1:**
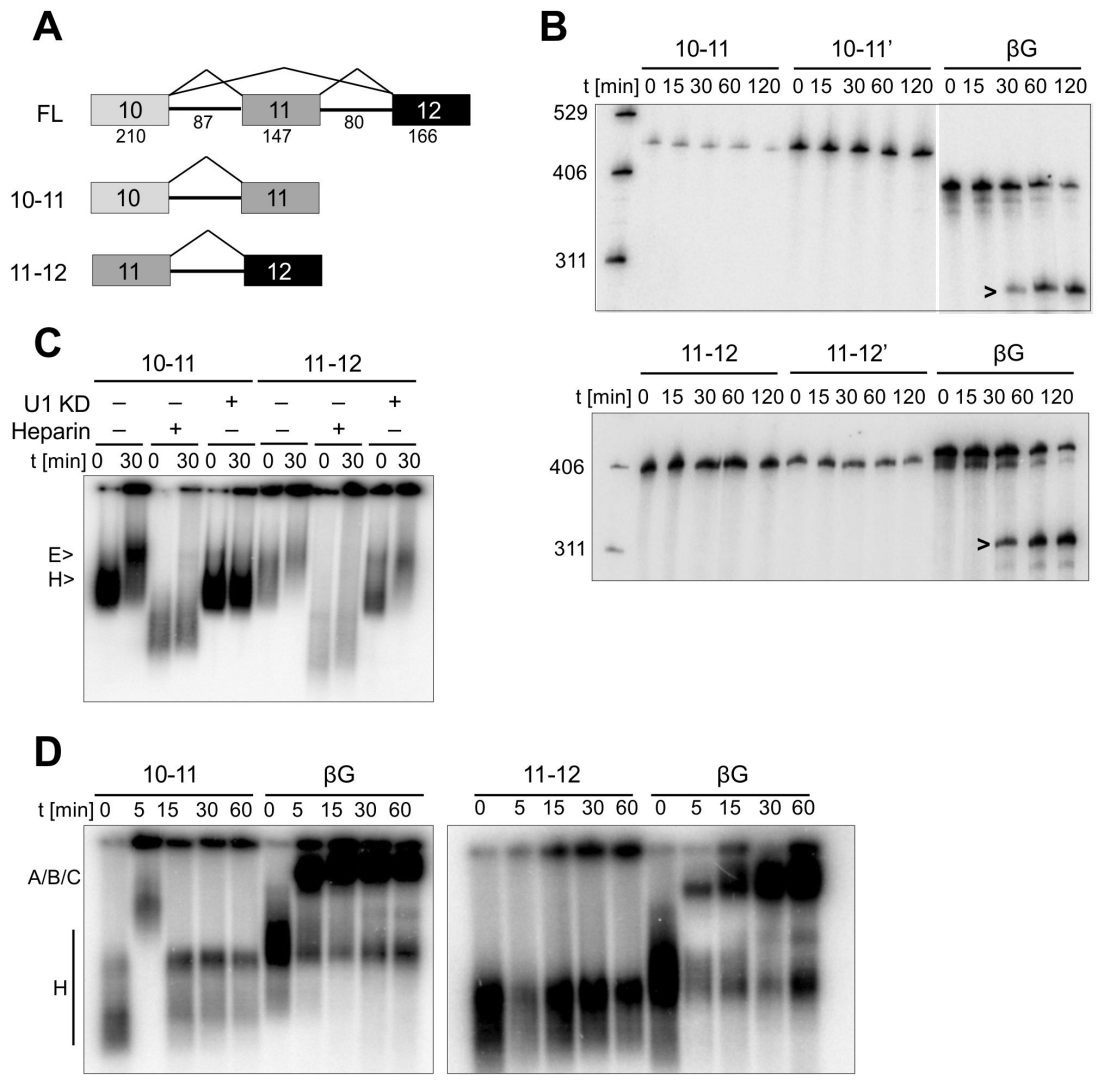
RON splicing and splicing complex formation *in* *vitro*. (A) Diagrams of RON pre-mRNA substrates and their expected patterns of splicing. Boxes represent RON exons 10 (210 nt), 11 (147 nt) and 12 (166 nt), and lines represent introns 10 (87 nt) and 11 (80 nt) respectively. (B) Time courses of splicing in HeLa nuclear extract of the single intron substrates. Pre-mRNA substrates 10-11 and 10-11’ begin at the 5’ end of exon 10, with an additional GGG for transcription initiation, and end at the 3’ end of exon 11 plus a 7 nt intron portion of either wild-type exon 11 5’ splice site sequence (11,12) or consensus 5’ ss sequence (GUAAGUU) (10-11’). Pre-mRNA substrates 11-12 and 11-12’ begin at nt 7 of exon 11, which forms a natural GGG tract, and end either at the end of exon 12 (11,12) plus 7 nt of the wild type 5’ splice site or with an additional 3’ consensus 5’ss (11-12’). βG pre-mRNA is derived from β-globin exons 2 (226 nt) and part of exon 3 (56 nt), with a reduced intron (106 nt). DNA marker lengths are as shown (nt). (C) Analysis by native gel electrophoresis of pre-mRNA-containing complexes following incubation under conditions permitting assembly of complex E for the times shown. Nuclear extract was pre-incubated where indicated (U1 KD) with a 2’-O-methyl oligonucleotide complementary to the 5’ end of U1 snRNA prior to addition of pre-mRNA or incubated with heparin following assembly. Spliceosomal complexes E and H are labeled to the left of the panel. (D) Native gel electrophoresis after incubation of pre-mRNA in nuclear extract under conditions permitting spliceosome assembly. Complex H and the spliceosomal complexes A, B, C are labeled to the left of the panel.

RON Δ165 mRNA has been identified in breast and gastric carcinoma tumours and has been directly linked to the development of an invasive phenotype in these tissues [25,26]. This isoform maintains the reading frame but lacks exon 11, which codes for the 49-amino acid trans-membrane region of the receptor (FL; Figure 1A). The loss of exon 11 results in incorrect processing of the receptor and localization to the cell cytoplasm. Homodimerization then occurs between the mis-localized receptors, due to incorrect disulphide bond formation, which results in constitutive activation [12,25,27].

Skipping of exon 11 and expression of RON Δ165 is stimulated by the binding of SRSF1 to RON exon 12 [19]. This is likely to be one route that mediates the known proto-oncogenic activity of SRSF1 [28]. Exon 11 skipping, SRSF1 expression and cell motility are regulated through phosphorylation of Sam68, which is a target of the ERK1/2 signalling pathway. The ERK signalling pathway is often itself miss-regulated in cancer and is, in addition, a signalling pathway downstream of RON [29,30]. Skipping of exon 11 is also stimulated by hnRNP H, which interacts with the 5’ end of exon 11 and correspondingly promotes cell invasiveness [31]. 

One of the striking features of splicing between exons 10, 11 and 12 of RON is that a variable but often high proportion of the RNA remains unspliced in both normal tissues and tumours [19]. Similarly high levels were seen when minigenes containing the genomic sequences from exon 10 to exon 12 were expressed in cell lines [19,31]. Indeed, unspliced RNA was by far the predominant product of one such minigene, and it remained so even when exon 11 inclusion was stimulated at the expense of skipping by knockdown of hnRNP H [31]. Such a preponderance of unspliced RNA is remarkable. One possible explanation is that splicing of RON pre-mRNA is suppressed specifically, as a mechanism for modulating the levels of expression of RON. The purpose of this research was to identify putative sites of such regulation and the mechanisms involved.

## Materials and Methods

### Construct synthesis

Mutants were constructed from overlapping PCR reactions done with Phusion DNA high fidelity DNA polymerase (Thermo Scientific) and cloned, after digestion with DpnI, into pCR®-Blunt II-TOPO® vector (Invitrogen). Following sequence confirmation, constructs were amplified for transcription by PCR. In each case the transcription template was generated using a forward primer containing a T7 polymerase promoter sequence, GGG to provide an efficient initiation site for transcription and a sequence complementary to the beginning of the first exon (nucleotide 1 of β-globin exon 2 and RON exon 10, or nucleotide 10 of RON exon 11). The reverse primer was complementary to the last exon of the construct. The 3’ end of each construct comprised the 3’ nucleotide of the 3’ exon, with the addition of the 5’ splice site consensus sequence, AGGTAAGTT, to the 3’ end of the RNA-sense strand of the transcription template. This applies to all constructs, with the exception of constructs 10-11 and 11-12 where the transcript ended at intronic position plus 6 of the wild-type 5’ splice site. The PCR products were purified and used as templates for *in vitro* transcription. Constructs with lengthened RON introns contained the 20 nt sequence 5’ AAA ATT CAT GTT ATA TGG TC 3’, which is found in the middle of the intron of the efficiently spliced β-globin-derived construct [32,33]. 

### Splicing in vitro

Transcription, purification, splicing and recovery of radioactive pre-mRNA was as described [32,34]. Splicing reactions were done in the presence of 3.2 mM MgCl_2_ and 100 mM potassium cations in HeLa nuclear extract (Cilbiotech). Radioactivity was detected and measured with a phosphorimager. Splicing complexes were analysed by native gel electrophoresis as described [35]. Complex assembly was stalled at complex E by omission of ATP, creatine phosphate and MgCl_2_ from reaction mixtures and pre-incubation at 30 °C for 30 min. Complexes E and A were identified by pre-incubation of splicing reaction mixtures with 2’-*O*-methyl oligonucleotides complementary to U1 and U2 and U6 snRNA, as appropriate [33].

### UV-crosslinking & immunoprecipitation

Crosslinking of proteins to pre-mRNA was done by irradiation for 5 min with a broad-wavelength UV source (SpotCure, UVP). After RNase treatment, samples were separated by SDS-PAGE and transferred electrophoretically to nitrocellulose. For identification of the crosslinked proteins by immunoprecipitation, crosslinking reactions were pre-cleared by incubation with protein G-agarose beads and then incubated with beads pre-bound to hnRNP H/F antibody (abcam: ab10689) or Living Colors full-length A.v. polyclonal antibody (Clonetech: 632460). After recovery and washing of the beads, the bound components were eluted in SDS, separated by SDS-PAGE and transferred to nitrocellulose. For the analysis of binding by U2AF65, transcripts were incubated in nuclear extract under conditions restricting assembly to formation of complex E, and U2AF65 and associated pre-mRNA was recovered on protein G-agarose beads pre-bound to an antibody specific for U2AF65 [36].

## Results

To determine the mechanisms by which regulatory factors affected the skipping of RON exon 11, splicing assays were done in nuclear extracts with a substrate comprising exons 10, 11 and 12 of RON (Figure 1A). The advantage of splicing *in vitro* is that the analysis of complexes and components can accompany tests on the functions of sequences. The splicing of RON was compared with a highly efficient transcript derived from β-globin [32,33]. However, the full-length transcript (FL) was unable to splice (data not shown). One possible reason for this was that the introns, of 87 and 80 nucleotides, were close to the minimum length found to splice well in HeLa nuclear extracts [37]. To test whether this was the case, the introns were expanded by insertion of 20 nts from a β-globin intron into the centre of introns 10 and 11, which brought them to approximately the same length as the highly efficient β-globin intron itself (106 nucleotides). However, no splicing was observed (data not shown). Substrates containing only a single intron and its flanking exons were also inactive (10-11 and 11-12, Figure 1B). Inclusion of a 5’ splice site (5’ss) at the 3’ ends of the transcripts can stimulate splicing [38], but there was no effect on the RON constructs (10-11’ and 11-12’, Figure 1B). To establish the stage at which spliceosome assembly was inhibited, complex formation was analysed under conditions permitting only the formation of complex E. Complex E is the first splicing-specific complex to form in nuclear extracts. It requires splice sites and it is committed to splicing, but it accumulates only in the near-absence of ATP [35,39-43]. It is distinguished by a requirement for U1 snRNPs, which base-pair to available splice sites [33], and its sensitivity to incubation with heparin before electrophoresis [35,39]. The short 10-11 pre-mRNA formed complex E, but it was not clear whether 11-12 did so also (Figure 1C). However, incubation with ATP and electrophoresis after addition of heparin showed that neither substrate could assemble pre-spliceosomal or spliceosome complexes (Figure 1D). We conclude that assembly is blocked prior to assembly of the pre-spliceosomal complex A.

### Sequences limiting RON Splicing

**Figure 2:**
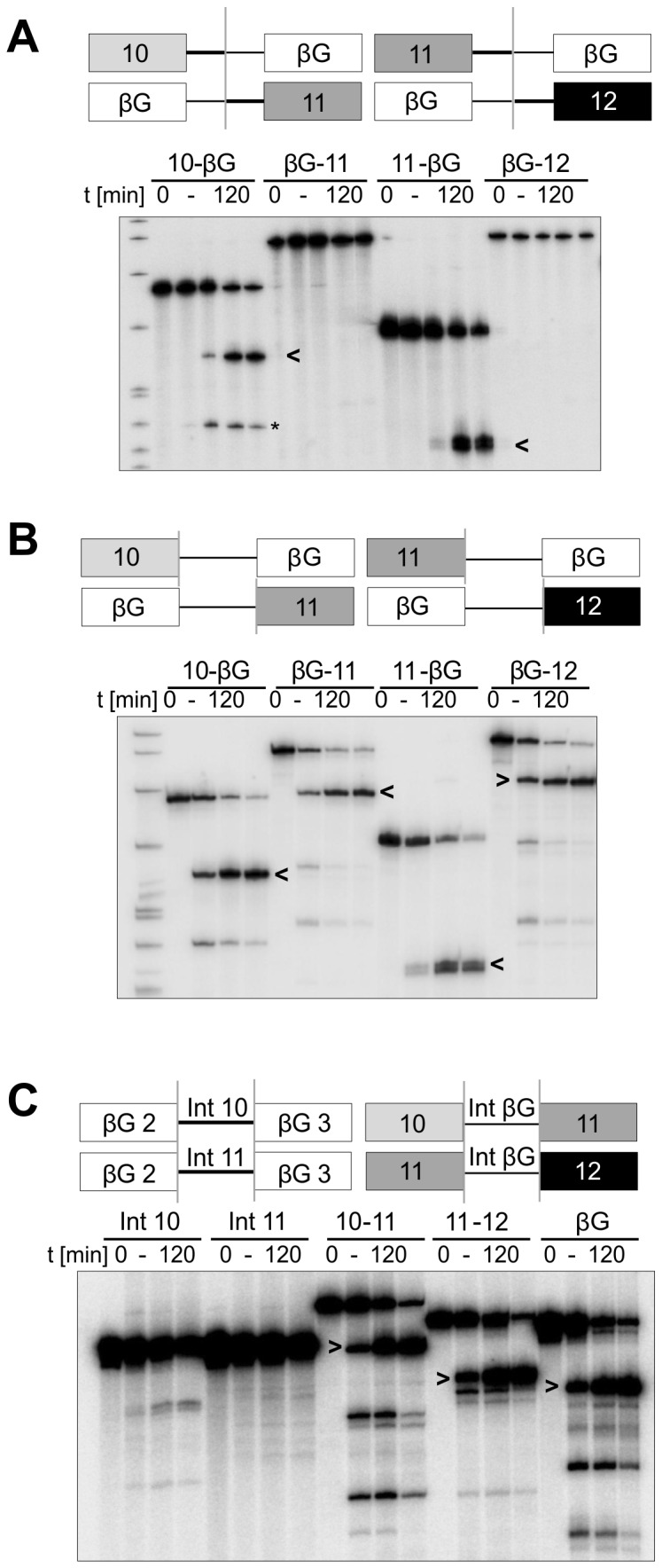
Splicing of RON/β-globin single intron hybrid transcripts. (A) Time courses of splicing *in* *vitro* of chimaeric transcripts comprising the 5’ or 3’ halves of RON 10-11 and 11-12 joined, as shown by the grey vertical lines, to the 3’ or 5’ halves of the β-globin 2-3. RON and β-globin introns are shown as thick and thin lines respectively. Arrowheads indicate the mRNA products. An asterisk (*) indicates the 5’ exon splicing intermediate of construct 10-β-Globin. Splicing reactions were incubated for 0, 15, 30, 60 or 120 min. Marker lengths (left-hand lane) in nt are 622, 527, 404, 309, 242, 238, 217, 201, and 190. (B) Time courses of splicing of transcripts in which RON exons 10, 11 or 12 exactly replaced β-globin exons 2 or 3, with the sites of fusion being at the exon-intron junctions (grey lines). Reactions were incubated for 0, 30, 60 or 120 min. (C) Time courses of splicing of transcripts containing RON introns 10 and 11 or the β-globin intron inserted between heterologous exons. Reaction times were as in (B).

A systematic search for sequences that prevent the splicing of RON exons 10, 11 and 12 was made by constructing hybrid single intron constructs with β-globin-derived sequences (Figure 2). The first series formed single intron constructs from fusions of RON sequences with either β-globin exon 2 and downstream intron sequences or β-globin exon 3 and upstream intron sequences (Figure 2A). The RON intron 10 sequence of 87 nts was divided to generate 43 nts of upstream and 44 nts of downstream intron sequence, plus neighbouring exons. The RON intron 11 sequence of 80 nts was divided into two equal halves. The β-globin intron was divided to generate 54 nts of upstream and 52 nts of downstream intron sequence (containing the branch site and polypyrimidine tract), plus neighbouring exons. Thus, the chimaeric introns had a total length of 92-98 nts, depending on the combination, which is intermediate between their natural lengths and the expansions by 20 nts, found as described above to have no effect. Constructs in which the RON exon formed the 5’ part (10-βG and 11-βG) spliced well, whereas with RON exons forming the 3’ part splicing was blocked (βG-11 and βG-12). However, when the RON sequences contributed only an exon, splicing was efficient regardless of whether RON contributed the 5’ or 3’ exon (Figure 2B). When the introns were tested in isolation, introns 10 and 11 either largely or completely prevented splicing of β-globin exons (Int10 and Int11, Figure 2C), whereas neither exons 10 and 11 nor exons 11 and 12 inhibited splicing of the β-globin intron. We infer that splicing was not affected by the RON exons, but that it was restricted by the 3’ part of both introns 10 and 11. 

**Figure 3:**
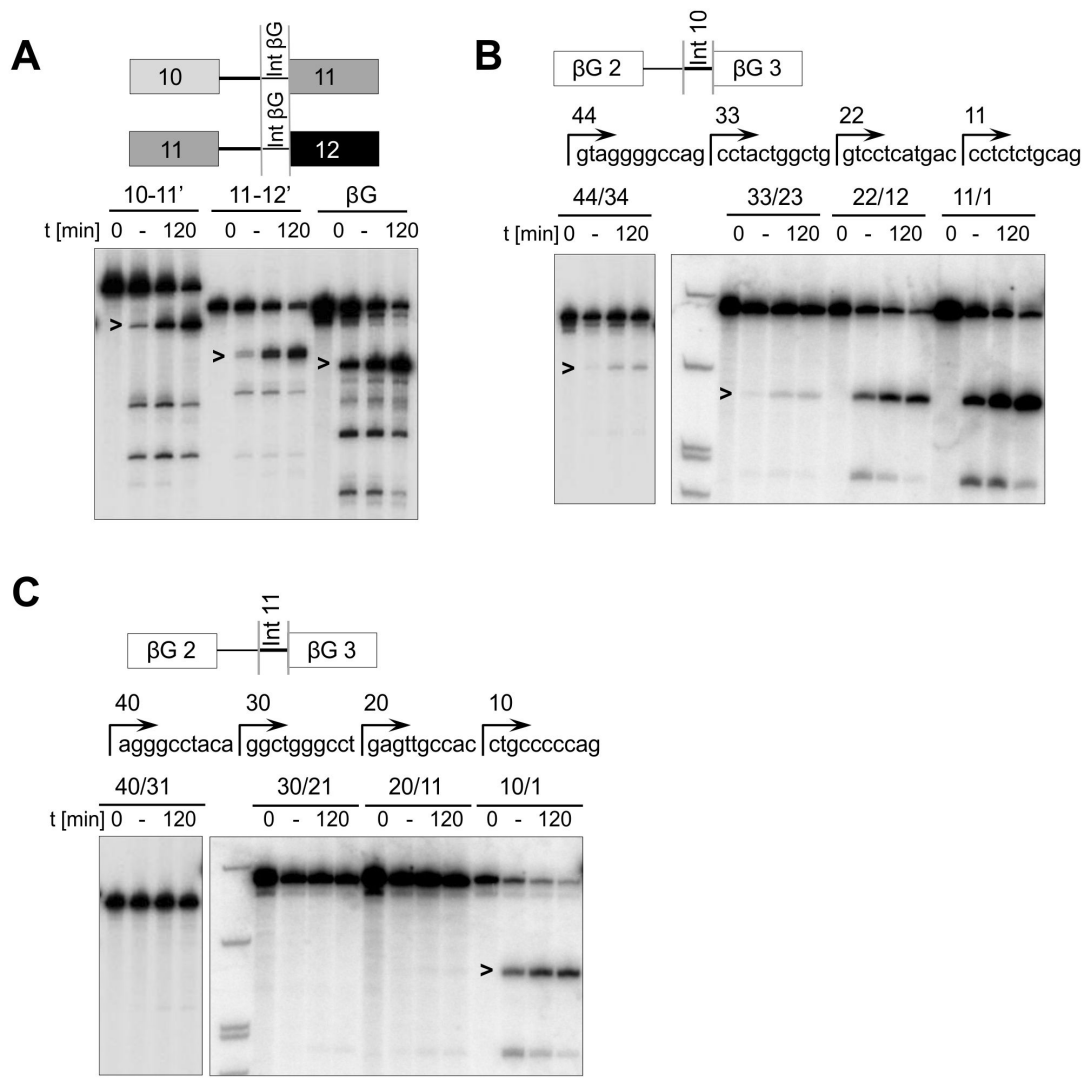
Identification of splice limiting sequences at the 3’ ends of RON introns 10 and 11. (A) Time courses of splicing of transcripts of RON 10-11 and 11-12 with a branchpoint sequence and polypyrimidine tract from β-globin. The RON transcripts contain a consensus 5’ss at the 3’ end. (B) Time courses of splicing of β-globin transcripts in which sequences from the 3’ end of RON intron 10 replaced corresponding portions of the 3’ end of the β-globin intron. The sequence of the 3’ end of RON intron 10 is marked to show the 5’-most extremity and the lengths of the RON sequences used. The transcripts are labelled with numbers showing the number of nt of RON included and the number of nt of β-globin replaced (RON/β-globin). (C) Reactions as in (B) but with transcripts containing portions of RON intron 11, as shown.

The inhibitory sequences were mapped more closely by further hybrids. Replacement of the 3’ 44 nts in RON 10-11’ and 40 nts in 11-12’ intron sequence with the 3’-most 52 nts of the β-globin intron sufficed to activate both constructs (Figure 3A). In contrast, replacing corresponding sequences in β-globin with the 3’-halves of introns 10 and 11, 44 nts and 40 nts respectively, inhibited splicing strongly (Figures 3B & 3C). To define the portions of the RON introns that inhibited the splicing of β-globin, the short RON sequences at the 3’ end of the β-globin intron were progressively replaced by the original β-globin intron. Splicing assays showed that splicing of the β-globin intron was reactivated when the length of the RON portion from intron 10 was reduced from 33 to 22 nts (Figure 3B). The total intron length was 98 nts in both cases. Significantly, the transition from 33 nts to 22 nts had reintroduced the branchpoint of β-globin, but the polypyrimidine tract was still contributed by RON intron 10. Thus, the polypyrimidine tract of RON intron 10 is not intrinsically inadequate, but any branchpoint function contributed by the 3’ 33 nts of intron 10 was very weak. The corresponding experiments with the 3’ part of intron 11 (Figure 3C) showed that 20 nts of intron 11 inhibited β-globin splicing, but 10 nts did not. The total intron size of the β-globin intron with RON intron 11 replacements of 40 nts, 30 nts and 20 nts was 97 nts, 100 nts and 103 nts respectively. In both cases the β-globin branchpoint was present, suggesting that either the distal part of the β-globin polypyrimidine tract is particularly important or that nucleotides -20 to -11 from the 3’ splice site (3’ss) of RON intron 11 contain an inhibitory sequence. The former possibility can be excluded, because the 22 nts of intron 10 supported splicing in the absence of the entire polypyrimidine tract of β-globin. A candidate inhibitory sequence in RON intron 11 is an AG dinucleotide at -19/-18 from the 3’ss. In summary, we conclude that intron 10 is not limited by its polypyrimidine tract, but that the branchpoint site or strength may cause poor splicing, whereas intron 11 is limited by inhibitory sequences in its polypyrimidine tract and the contribution of its branchpoint is unknown.

**Figure 4:**
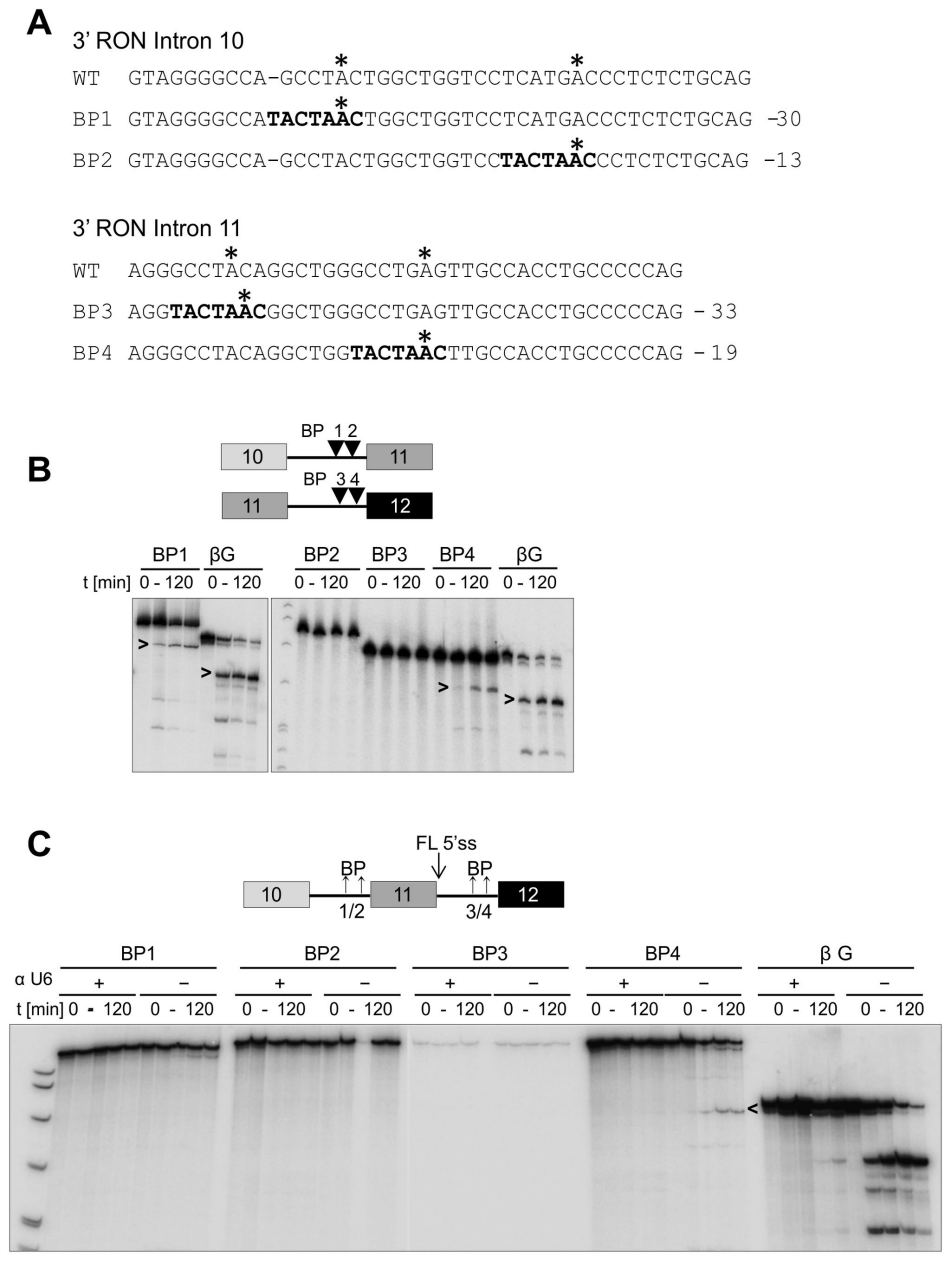
Testing the ability of consensus branchpoint sequences to enable RON splicing *in* *vitro*. (A) Sequences of the 3’ halves of RON introns 10 and 11 showing the introduced branchpoint sequences (bold). Possible branchpoints are highlighted by asterisks. (B) Time courses of splicing of single intron RON transcripts with consensus branchpoints: 10-11 (BP1 & BP2) and 11-12 (BP3 & BP4). (C) Splicing time courses of FL RON transcripts containing both introns and single branchpoint mutations. Reactions were done in the presence (+) or absence (-) of an inhibitory 2’-O-methyl oligonucleotide complementary to U6 snRNA.

The contributions of the branchpoints were tested by direct mutagenesis. Two candidate branch sites in each intron were replaced by a consensus branchpoint sequence (UACUAA*C, where A* is the branchpoint; Figure 4A). In intron 10, a branchpoint at position -30 (BP1) relative to the 3’ss enabled some weak splicing activity, whereas at -13 (BP2) it was inactive (Figure 4B). In intron 11, introducing a consensus branchpoint at position -33 (BP3) had no effect, whereas at -19 (BP4) it produced a very low level of splicing. The same mutations were introduced into the full-length 10-11-12 transcript (Figure 4C). The consensus branchpoint at -19 relative to the 3’ss of intron 11 enabled a very low level of skipped mRNA to be produced, but the other mutations were ineffectual. For intron 10, we conclude that the branchpoint is so weak that it cannot support splicing of a β-globin intron, but splicing of intron 10 requires improvements to the polypyrimidine tract as well as an improved branchpoint (c.f. Figure 3A). For intron 11, the 3’ss-distal part of the polypyrimidine tract is sufficient to block β-globin splicing, possibly because an AG dinucleotide at the most likely site of its branchpoint inhibited the use of the β-globin branchpoint; while splicing of intron 11 itself is also limited by more than just the branchpoint, an improved branchpoint is sufficient for some splicing of the full-length 10-11-12 pre-mRNA.

### Protein binding to the limiting sequences

**Figure 5:**
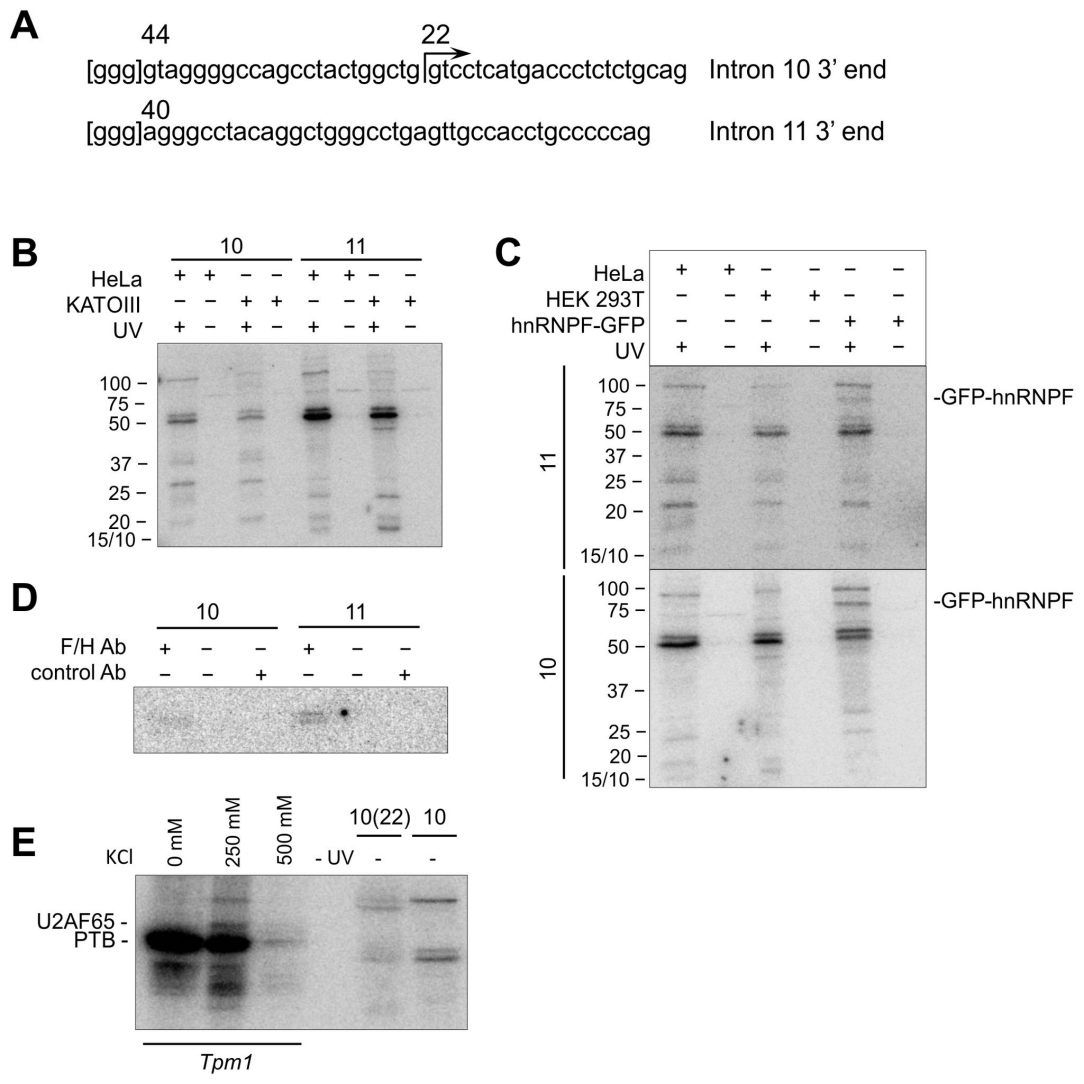
Analysis of proteins contacting the limiting sequences of RON introns 10 and 11 by crosslinking. (A) Sequences of transcripts used for UV-crosslinking. These comprised 44 nt and 40 nt from the 3’ ends of intron 10 and 11, respectively, together with a 5’GGG added to enhance transcription. In the natural sequence a GGG motif is found 4 nts and 2 nts 5’ of these intron 10 and 11 sequences, respectively. The start of a shorter intron 10 transcript of 22 nt is shown. (B) Detection by UV-crosslinking and SDS-PAGE of proteins bound to [α-^32^P]GTP-labelled transcripts of the 44 nt and 40 nt sequences of introns 10 and 11. Control samples were not irradiated (UV -). Transcripts were incubated in nuclear extracts from HeLa and KATOIII cells. The extent of migration of protein size markers is shown. (C) UV-crosslinking analysis as in (B) to the 44 nt and 40 nt transcripts of introns 10 and 11 after incubation in nuclear extracts from HeLa and HEK293T cells and from HEK293T cells expressing GFP-hnRNP F. (D) Immunoprecipitation of products from UV-crosslinking in HeLa nuclear extract with anti-hnRNP F/H. (E) UV-crosslinking to detect binding of U2AF65 or PTB. Transcripts were labelled by inclusion of [α-^32^P]UTP and [α-^32^P]GTP during transcription. Transcripts were incubated in HeLa nuclear extract and processed as in panel B. The sequences used comprised the 44 nt and 22 nt portions of intron 10, as in (A). As markers for the two proteins, crosslinking was done also with a portion of *Tpm1* 5’ of exon 3 that crosslinks readily to PTB at low salt concentrations [49] and, at higher concentrations, to U2AF65 (C. Gooding, personal communication).

The inhibition of β-globin splicing by insertion of portions of the 3’ splice site regions might result either from the inadequacy of the splicing signals or from the binding of inhibitory proteins, such as hnRNPs. The possible binding of proteins other than splicing factors to these regions was examined by UV-crosslinking to transcripts encompassing the 3’-most 44 and 40 nts of introns 10 and 11, respectively (Figure 5A). Figure 5B shows crosslinking to [α-32P]GTP-labelled transcripts in nuclear extracts from HeLa cells, used for all the splicing assays. As a control to test whether the interactions detected might reveal only constitutive factors rather than any factors that specifically repress exon 11 inclusion, we also tested nuclear extracts from KATOIII cells, which naturally express the Δ165 isoform [19]. The results were identical. The conspicuous doublet of proteins at ~50 kDa was assigned to hnRNP F/H for three reasons: (i), the sequences contained a number of GGG motifs, known to be the core element in hnRNP F/H binding sites [44,45], including a 5’ GGG sequence incorporated to maximize the efficiency of transcription; (ii), crosslinking in an extract from HEK293T cells expressing a GFP-hnRNP F fusion protein produced a novel band of an appropriate size (Figure 5C), demonstrating that hnRNP F could bind, and moreover in the case of intron 11 this was clearly at the expense of one of the 50 kDa bands; (iii), a protein doublet of ~50 kDa was immunoprecipitated after crosslinking with a monoclonal antibody to hnRNP F/H (Figure 5D). The hnRNP F/H proteins are not candidate inhibitors, however, since their G-tract targets lie outside the 3’ 33 nts of intron 10 or the 3’ 20 nts of intron 11 that mediate inhibition (Figure 3B). Strikingly, however, crosslinking to transcripts labelled with [α-32P]UTP and [α-32P]GTP (Figure 5E) showed no evidence for binding by PTB, a common repressor [46], nor by U2AF65, the splicing factor that normally binds the polypyrimidine tract [47,48], both of which can be assigned by the position of the crosslinks formed with a substrate based on *Tpm1* exon 3 [49]. The same results were obtained with transcripts labelled solely with [α-32P]UTP (not shown). The results suggest that the limiting efficacy of these sequences is more likely to be the result of deficient binding by constitutive factors than specific binding by inhibitors.

### 3’ splice site recognition

**Figure 6:**
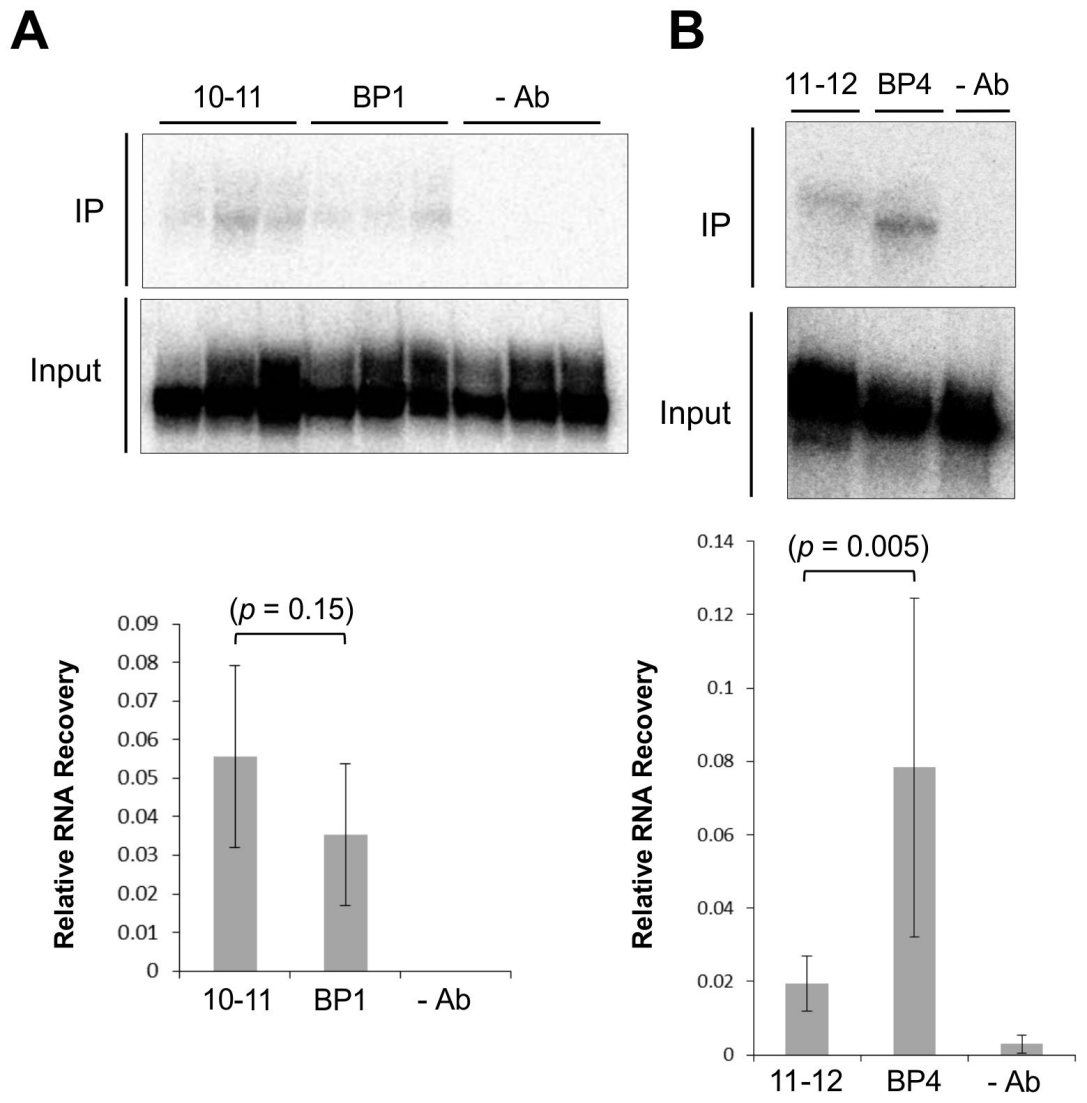
Effects of branchpoint consensus sequences on U2AF65 binding to RON introns 10 and 11. (A) Immunoprecipitation of RON 10-11 and BP1 RNA with anti-U2AF65 after incubation in HeLa nuclear extract. The immunoprecipitated and input RNA was analysed by gel electrophoresis. The radioactivity was quantified with a phosphorimager. Reactions were done in triplicate and the mean proportion of input RNA precipitated is shown, together with the sample standard deviations. The probability, p, that the samples are from the same population was calculated by Student’s t. (B) Immunoprecipitation as above with RON 11-12 and BP4 RNA. The experiment was done five times. On four of these the levels of immunoprecipitated RNA were above background. The mean proportions of input RNA precipitated in these four experiments are shown as above. The calculation of the probability that the samples 11-12 and BP4 do not differ was calculated using a ratio t test.

In both intron 10 and intron 11, efficient splicing was achieved only when both the branchpoint sequences and the polypyrimidine tracts were replaced (Figure 3A), even though the effects when these sequences were introduced in β-globin suggested that the deficiencies of the branchpoint were most acute. The interdependence of these elements is consistent with observations that U2AF65 normally forms a complex with the branchpoint binding protein SF1 at an early stage in complex assembly, enabling their binding to the polypyrimidine tract and branchpoint respectively [50-52]. If U2AF65 binding was limiting, then the slight improvement in splicing seen when the branchpoints were mutated to the consensus sequence would be accompanied by an increase in U2AF65 binding. This was tested by immunoprecipitation of the wild-type and branchpoint-upregulated versions of 10-11 and 11-12 (mutants BP1 and BP4, Figure 4). The pre-mRNAs were incubated in nuclear extract depleted of ATP and immunoprecipitated with an antibody to U2AF [36]. Both pre-mRNAs were immunoprecipitated (Figure 6). The apparent contradiction with the failure to detect crosslinking (Figure 5) might be explained either by the increased sensitivity when the whole pre-mRNA is detected by immunoprecipitation or by the ability of U2AF65 to bind non-specifically and to sites other than polypyrimidine tracts at 3’ splice sites [53,54]. The recovery of 10-11 pre-mRNA was not significantly affected by the presence of a consensus branchpoint (Figure 6A), whereas in several independent experiments a consensus branchpoint did enhance the recovery of 11-12 pre-mRNA (Figure 6B). It is possible that the polypyrimidine tract of intron 11 is more dependent on the branchpoint sequence.

## Discussion

The purpose of these experiments was to investigate the mechanisms underlying the poor splicing of RON exons 10-11-12 in endogenous RNA and in transfected minigenes [19,31]. We had originally thought that the poor splicing of RON *in vivo* might have some functional importance, either as a means of regulating RON expression or as a by-product of mechanisms that facilitate exon skipping. One possibility was that this effect was connected with the unusually short introns, which contain multiple G-rich tracts and might act as targets for hnRNP F/H, as has been reported for exon 11 itself [31]. While hnRNP F/H binding and G-triplets in introns are both known to stimulate splicing, we had wondered whether the effects might be different with such short introns.

Our initial results showed that the RON exons 10, 11 and 12 did not splice *in vitro* under conditions in which a standard substrate was spliced efficiently. In this respect, splicing *in vitro* recapitulated or even magnified the *in vivo* effect, whether it resulted from poor intrinsic reactivity or suppression. This did not result from interactions between the two introns, as each intron separately was unable to splice. The use of single intron constructs showed that the effects were not caused by repression of the exons, which was surprising in view of the suppression of exon 11 inclusion by proteins binding to exons 11 or 12 [19,31]. Expansion of the introns to bring them up to the length of the efficiently spliced control intron did not activate splicing. Moreover, a systematic use of chimaeric introns showed that in both introns the portions containing the G-rich tracts did not have a dominant inhibitory role, excluding any role for hnRNP F/H. Instead, sequences in the region of the polypyrimidine tract and branchpoint were limiting. Protein crosslinking and immunoprecipitation showed that the binding of U2AF65 was very weak. Interestingly, given that improving the intron 11 branchpoint enabled some splicing of the full-length construct, this mutation appeared to improve the binding of U2AF65 to the pre-mRNA. 

These findings suggested that the deficiency in splicing arises from low levels of binding by constitutive factors to the branchpoint and polypyrimidine tract, although we cannot exclude the possibility that some of the proteins that showed low levels of crosslinking might also have an effect. Limiting levels of 3’ splice site binding by U2AF65 and U2 snRNPs, either together or separately, have been linked to skipping of a number of exons, including *SMN2* exon 7, *Fas* exon 6 and *RB1* exon 9 [55-58]. This appears to be incompatible with the observation that complex E formed on intron 10, which appears to indicate that U2AF65 had bound. However, a similar complex to E (E’) can form in the absence of U2AF [59]. Although it contains the branchpoint-binding protein SF1, it does not require either the polypyrimidine tract or the branchpoint sequence for its formation. It is possible, therefore, that the complex seen on the gels is E’. 

The finding that neither the introns’ lengths nor their high G content contribute to deficient splicing is consistent with published data, despite the fact that 95% of human introns are longer than 100 nts [60]. The peak in the distribution of intron sizes at nucleotide resolution is, in fact, at 87 nts [60]. A recent analysis of 179 human genome sequences has suggested that there is a strong selection for a minimal intron size of 87 nts, the length of RON intron 10, and it is characteristic of such short introns that their (G+C) content is either equal to or higher than that of the flanking exons [61]. Such a nucleotide distribution is in marked contrast to that of exons flanked by longer introns, where there is a strong asymmetry in nucleotide composition. Indeed, it has been suggested that the asymmetry is linked to splicing by exon definition, whereas the short, (G+C)-rich introns are associated with intron definition mechanisms [62]. Interestingly, our crosslinking studies suggest that such introns will be bound by hnRNP F/H. Whereas conventional proposals suggest that short introns are defined by highly efficient interactions between the ends of the intron, being in close proximity, it might be that the ends of the introns are marked by the pattern of hnRNP F/H binding and that a high (G+C) content is therefore required for the splicing of short introns.

A further aspect of the intron length is worth noting. The architecture of this region does not appear to have evolved as might have been expected for biologically selected exon skipping. Exon skipping is associated with weak splice sites, short exons and long flanking introns [63,64]. Instead, intron definition processes associated with short introns are likely to promote constitutive inclusion. The development of weak branchpoint and polypyrimidine tract signals might have been driven as a route by which exon skipping could be promoted where intron definition occurs. In other words, where the architecture of the gene strongly favours splicing between the ends of short introns, it is possible that inefficient splicing signals and therefore inefficient splicing are tolerated if there are selective advantages in exon skipping.

A final issue is the means by which splicing *in vivo* happens at all, given that we see no splicing *in vitro*. It is known that splicing is more efficient when coupled to transcription [39]. However, it is not clear why this should be so, in the absence of clear evidence for functional effects arising from direct mechanistic connections between splicing components and RNA polymerase [65,66]. One possibility is that secondary or other structures form in RNA transcribed *in vitro* that do not form *in vivo* because hnRNP proteins bind before they form [67,68]. As noted above, introns 10 and 11 contain a number of G-triplets that could bind hnRNP F/H [44,69]. They could also form quadruplex structures, which form at relatively low rates but are stable once formed [70,71]. It is possible that transcription *in vitro* leads to the formation of quadruplexes that prevent hnRNP binding when the RNA is added to nuclear extract, whereas *in vivo* hnRNP F/H might bind first. The effects of this on the efficiency of splicing are speculative, but we note that there is evidence that the binding of hnRNP H facilitates recruitment of U2AF65 [72]. Thus, although splicing *in vivo* is inefficient, co-transcriptional binding of hnRNP H and recruitment of U2AF65 might compensate to some extent for the intrinsic weakness of the branchpoint and polypyrimidine tracts.
